# An Incidental Finding of Whipple's Disease Masquerading as Nonspecific, Long-Standing Symptoms

**DOI:** 10.1155/crdi/3177799

**Published:** 2024-12-27

**Authors:** Thomas Tuggle, Alison I. Orvin, Christian Caveness, Christopher Ingram

**Affiliations:** ^1^Department of Pharmacy, WakeMed Health and Hospitals, 3000 New Bern Ave, Raleigh 27610, North Carolina, USA; ^2^Department of Pharmacy Services, Carilion Clinic, 1906 Belleview Ave, Roanoke 24014, Virginia, USA; ^3^Division of Practice Advancement and Clinical Education, University of North Carolina Eshelman School of Pharmacy, 301 Pharmacy Lane, Chapel Hill 27599, North Carolina, USA; ^4^WakeMed's Division of Infectious Diseases, WakeMed Health and Hospitals, 3000 New Bern Ave, Raleigh 27610, North Carolina, USA

**Keywords:** gastrointestinal infection, incidental finding, *Tropheryma whipplei*, Whipple's disease

## Abstract

Whipple's disease is a rare bacterial infection that is often present for years prior to diagnosis. Symptoms are nonspecific in the early stages of presentation and are primarily gastrointestinal in nature. The disease may progress with more systemic symptoms including arthralgia, fever, lymphadenopathy, cardiovascular disease, and central nervous system involvement. This case describes a man with a history of long-standing, nonspecific symptoms who only began to show significant improvement after an incidental finding of Whipple's disease. Due to its rare nature, other instances of the disease have likely gone undiagnosed. A brief review of relevant literature is also included.

## 1. Introduction

Whipple's disease is a rare bacterial infection caused by the intracellular gram-positive rod *Tropheryma whipplei.* The most common presentation is gastrointestinal illness causing abdominal pain, malabsorption, diarrhea, and weight loss. Rarely, Whipple's disease is implicated in central nervous system (CNS), joint, vascular, and cardiovascular disease, causing systemic symptoms such as low-grade fever, lymphadenopathy, arthralgia, pleuritis, and other severe manifestations [1,2]. Though many patients may be carriers for the bacteria, the disease is extremely rare with an estimated yearly incidence of one to six in ten-million patients, with the majority of those affected being of American and European descent [3]. Nonspecific symptoms and vague clinical presentation may delay an accurate diagnosis as patients often first report symptoms 5  to 7  years before a diagnosis is made [4, 5]. If left untreated, Whipple's disease can become a severe, life-threatening infection with a mortality rate of up to 16.7% [6]. Treatment of alternative diagnoses, such as rheumatological diseases, with immunosuppressive therapies has been linked to increased harm in several reports [7]. These factors make diagnosis of Whipple's disease imperative for patient resolution of symptoms, reduction of disease progression, and prevention of unnecessary therapies. This case includes a patient with a history of long-standing, nonspecific symptoms further complicated by a lengthy past medical history.

## 2. Case

An 80-year-old Caucasian male presented with dizziness, generalized weakness, fatigue, and increased diarrhea over the previous three days. The patient reported a long-standing history of shoulder, wrist and back pain, chronic diarrhea, night sweats, and an approximate one-hundred-pound weight loss over recent years. Other past medical history included prostate cancer status post prostatectomy 17 years ago, hypertension, hyperlipidemia, macular degeneration, gastroesophageal reflux disease, antiphospholipid syndrome, and paroxysmal atrial fibrillation. The patient reported an allergy of hives to sulfonamide antibiotics. On presentation, the patient was afebrile, tachycardic (107 beats per minute), and hypotensive (96/61 mmHg) with a leukocytosis (14.5 K/μL). He was placed on empiric anti-infective therapy with ceftriaxone 2 g intravenous (IV) daily and metronidazole 500 mg IV three times daily and admitted to the internal medicine primary service for further evaluation.

Initially concerned for gastroenteritis, a *Clostridioides difficile* toxin and antigen assay, as well as a gastrointestinal pathogen panel were ordered, both of which detected no targets. An abdominal computerized tomography scan was performed and showed no acute abnormalities. Initial sets of blood cultures, as well as a second set 5 days later returned no growth. Human immunodeficiency virus serology was negative. The patient underwent a transthoracic echocardiogram that detailed mild thickening of the aortic valve, moderate mitral annular calcifications and thickening of the leaflets, and mild regurgitation. The C-reactive protein obtained on day 2 of admission was elevated at 12.51 mg/dL. After 6 days without improvement, an increased leukocytosis to 21.4 K/μL and progressive anemia (initial hemoglobin 10.0 g/dL, nadir 6.7 g/dL), an esophagogastroduodenoscopy (EGD) and colonoscopy were performed to rule out a gastrointestinal bleed.

**Figure 1:**
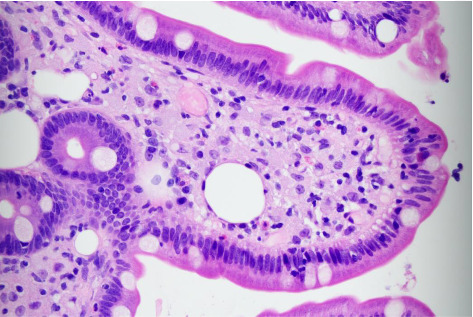
Duodenal biopsy, high power. Villus with expansion of the lamina propria by histocytes and lipid deposits.

**Figure 2:**
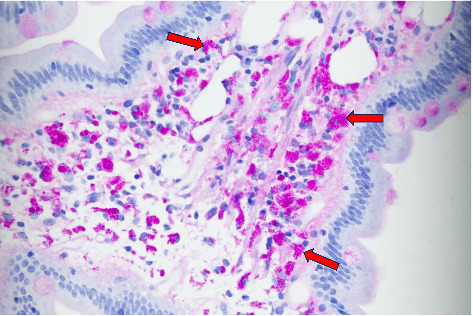
Duodenal biopsy, high power. Tightly clustered, rod-shaped bacteria, stained PAS-positive (bright pink in color). *T. whipplei* bacteria are typically 1–2.5 μm in length and 0.2–0.25 μm in width [8].

The EGD showed esophagitis with a white lesion and scalloping in the duodenal folds. Colonoscopy findings were significant for a sessile polyp in the sigmoid colon that was removed. Biopsies were performed on lower esophageal, gastric, duodenal, gastric, ileal, cecal, and sigmoid samples (Figure 1). All samples reviewed were negative for *Helicobacter pylori* and Barrett's esophagus. The lower esophagus biopsy did reveal a pattern of chronically inflamed gastric mucosa. Both the duodenum and terminal ileum biopsy showed lamina propria macrophage infiltrate containing periodic acid-Schiff (PAS) positive material, as well as histocyte and lipid deposits (Figure 2). Based on the histological findings of PAS-positive macrophages concerning for Whipple's disease, a confirmatory polymerase chain reaction (PCR) test was performed on the suspected tissue which returned positive for *Tropheryma whipplei*. Infectious diseases were then consulted for further management. Due to the association of Whipple's disease with CNS infection and endocarditis, the patient was initiated on ceftriaxone 2 gm twice a day IV for 4 weeks. After IV therapy, the patient was placed on doxycycline 100 mg twice a day and hydroxychloroquine 200 mg three times a day by mouth with a planned duration of 1 year. Following 1 month of treatment, the patient endorsed significant improvement in his arthralgia and chronic diarrhea. He had also regained a small amount of weight. However, no significant improvements in anemia were noted. At 6 months follow-up the patient continued to show slow improvements in his energy and arthralgia, and resolution of sweats, nausea, and diarrhea. He has also remained weight neutral since diagnosis.

## 3. Discussion

Because of its low incidence, Whipple's disease is typically not at the forefront of a differential diagnosis. Time to diagnosis may be delayed, leaving patients experiencing chronic symptoms [4]. The multiphasic pathophysiology of Whipple's disease occurs over several years and may contribute to this delayed diagnosis. In the early phase, patients may be asymptomatic; the most prevalent symptoms are nonspecific consisting of arthralgia, fever, fatigue, anemia, and lymphadenopathy [9]. The disease may progress to a second phase, with patients exhibiting gastrointestinal and CNS symptoms after an average latency period of six to 8 years. These more severe symptoms may prompt patients to present for treatment and receive a diagnosis [10].

As seen in our patient, initial suspicion for *Tropheryma whipplei* often comes from histological samples obtained during EGD and colonoscopy procedures. Positive histological signs show foamy macrophages, histocyte, and lipid deposits, and are often reactive to PAS reagents [11,12]. Confirmatory diagnostic testing is typically confirmed through PCR of duodenal tissue biopsy samples with a sensitivity of up to 96% [13]. Both the PAS stain and the PCR test are the gold standard in diagnostic confirmation for Whipple's disease. This highlights the importance of endoscopy with subsequent histological evaluation as necessary for identification of Whipple's disease [12].

The mainstay of treatment consists of antibiotic therapy. For initial therapy, use of IV ceftriaxone, penicillin G, or meropenem is recommended [14]. The maintenance phase of therapy consists of one double-strength tablet of trimethoprim-sulfamethoxazole given orally twice daily, with an alternative regimen of doxycycline 100 mg orally twice a day in combination with hydroxychloroquine 200 mg orally two to three times a day. The doses and duration of antibiotic therapy are specific to the type of disease, with CNS infection and endocarditis requiring longer durations of initial therapy [14,15]. Our patient was given several days of effective antibiotics prior to the diagnosis of Whipple's disease, preventing a clear diagnosis of CNS disease. Nonetheless, CNS manifestations could not be excluded as our patient had presented with dizziness, which can be seen in CNS infections [16]. Patients may respond quickly to treatment, though if left undiagnosed and untreated, the prognosis becomes poor. Dissemination into the CNS is regarded as the most severe form of the disease, with high risk of relapse and long-lasting neurological sequela. CNS infection could not be excluded, so more aggressive treatment was utilized for this case [5,17].

Whipple's disease patients, including the one described here will require close follow-up and monitoring for a significant period. Common risk factors for relapse include later presentation, disease with gastrointestinal symptoms, male, and a delayed diagnosis [18]. Due to acquired antimicrobial resistance, treatment failures may also occur, as genetic studies have shown development of resistance over the course of therapy [19]. These difficulties in treatment require continued patient care in the outpatient setting well after the diagnosis is made.

This patient is no exception to the often nonspecific and vague clinical picture of Whipple's disease. The diagnosis was made largely through incidental findings and workup for additional conditions on the differential diagnosis. The finding was paramount to the patient's overall recovery. Without the diagnosis, the initial antibiotics may have provided some acute relief symptoms, but without the prolonged duration of therapy required for appropriate Whipple's treatment, likely would have resulted in eventual relapsing disease, or disease progression for the patient. The intricacies of Whipple's disease, marked by the wide array of clinical presentations, emphasize the importance of a comprehensive and thorough approach. In cases where patients have chronic gastrointestinal and inflammatory symptoms without an identifiable cause, pursuing a differential diagnosis of Whipple's disease may be beneficial.

## Data Availability

The data used and/or analyzed during this study are available from the corresponding author upon reasonable request.
